# Dimensional structure of one-year post-COVID-19 neuropsychiatric and somatic sequelae and association with role impairment

**DOI:** 10.1038/s41598-023-39209-z

**Published:** 2023-07-27

**Authors:** Owen N. W. Leung, Nicholas K. H. Chiu, Samuel Y. S. Wong, Pim Cuijpers, Jordi Alonso, Paul K. S. Chan, Grace Lui, Eliza Wong, Ronny Bruffaerts, Benjamin H. K. Yip, Philippe Mortier, Gemma Vilagut, Dora Kwok, Linda C. W. Lam, Ronald C. Kessler, Arthur D. P. Mak

**Affiliations:** 1 Department of Psychiatry, Faculty of Medicine, The Chinese University of Hong Kong, Hong Kong, Hong Kong; 2 Jockey Club School of Public Health and Primary Care, The Chinese University of Hong Kong, Hong Kong, Hong Kong; 3 Department of Clinical, Neuro and Developmental Psychology, Amsterdam Public Health Research Institute, Vrije Universiteit Amsterdam, Amsterdam, The Netherlands; 4 International Institute for Psychotherapy, Babeș-Bolyai University, Cluj-Napoca, Romania; 5 Health Services Research group, IMIM-Institut Hospital Mar d’Investigacions Mèdiques, Universitat Pompeu Fabra, CIBERESP, Barcelona, Spain; 6 Health Services Research group, IMIM-Institut Hospital Mar d’Investigacions Mèdiques, CIBER Epidemiología y Salud Pública CIBERESP, Barcelona, Spain; 7 Department of Microbiology, Faculty of Medicine, The Chinese University of Hong Kong, Hong Kong, Hong Kong; 8 Department of Medicine and Therapeutics, Faculty of Medicine, The Chinese University of Hong Kong, Hong Kong, Hong Kong; 9 Universitair Psychiatrisch Centrum - Katholieke Universiteit Leuven (UPC-KUL), Campus Gasthuisberg, Leuven, Belgium; 10 Department of Health Care Policy, Harvard Medical School, Boston, MA USA

**Keywords:** Medical research, Epidemiology

## Abstract

This study examined the latent structure of the broad range of complex neuropsychiatric morbidities occurring 1 year after COVID-19 infection. As part of the CU-COVID19 study, 248 (response rate=39.3%) of 631 adults hospitalized for COVID-19 infection in Hong Kong completed an online survey between March-2021 and January-2022. Disorder prevalence was compared against a random non-infected household sample (n=1834). 248 surveys were received on average 321 days post-infection (Mean age: 48.9, 54% female, moderate/severe/critical infection: 58.2%). 32.4% were screened to have at least one mental disorder, 78.7% of whom had concurrent fatigue/subjective cognitive impairment (SCI). Only PTSD (19.1%) was significantly more common than control (14%, p=0.047). Latent profile analysis classified individuals into P1 (12·4%)-no current neuropsychiatric morbidities, P2 (23.1%)-SCI/fatigue, P3 (45.2%)-anxiety/PTSD, P4 (19.3%)-depression. SCI and fatigue pervaded in all profiles (P2-4) with neuropsychiatric morbidities one-year post-infection. PTSD, anxiety and depressive symptoms were most important in differentiating P2-4. Past mental health and P4 independently predicted functional impairment. Neuropsychiatric morbidity was associated with past mental health, reduced resilience, financial problems, but not COVID-19 severity. Their confluence with depressive and anxiety symptoms predicted impairment and are associated with psychological and environmental factors.

## Introduction

Recent studies found that physical and neuropsychiatric symptoms of ‘Post-COVID-19 syndrome’ (PCS, or “Long COVID”) including fatigue, cognitive impairment, anxiety, and depression^1^, may remain impairing 1 year^2^ after acute respiratory syndrome coronavirus 2 (SARS-CoV-2) infection. The nature and inter-relationships among these inherently overlapping clinical phenomena enduring after COVID-19 infection remain unclear, belying major knowledge gaps. First, few studies examined the symptom structure of anxiety and depression^2^ in COVID-19 survivors in sufficient granularity to determine their association with post-traumatic stress^3^, adversities shared with non-COVID-19 populations, and post-infectious psychophysiological changes^4^. Second, few studies examined the complex associations among fatigue, cognitive impairment, anxiety, and depression. Around one-third of COVID-19 survivors reported fatigue^5,6^, which may be a manifestation of post-COVID-19 pro-inflammatory changes^7^, chronic fatigue syndrome^8^ or depression, and anxiety^9^, all of which are common in COVID-19 survivors. One-fifth of COVID-19 survivors had subjective and objectively-measured cognitive impairment^5,10^, which had been described in post-viral encephalopathies, functional neurological disorders^11^, anxiety, and depression. Recent studies found cognitive impairment weakly connected to fatigue and neuropsychiatric symptoms^12^, a subset of post-COVID-19 cognitive-impaired individuals had elevated pro-inflammatory markers^5^, indicating heterogeneity in the nature of post-COVID-19 cognitive problems. Third, the extent to which psychiatric, cognitive, and somatic symptoms may contribute to role impairment remains ambiguous^6^, with public health implications in directing resources to treating conditions causing the greatest societal burden.

Various dimension reduction and clustering approaches have been deployed to explore the relationship amongst overlapping post-COVID19 clinical phenomena. Principal component analysis (PCA) on cognitive performance and clinical data in 46 adults 6 months post-COVID infection^13^ found cognitive test performance associated with acute infection severity but not with post-COVID mental health. PCA however could not reveal multivariate symptom profiles that are better identified using methods that allow for higher-order associations among symptoms. Using one of the latter methods, K-medioid clustering, the PHOSP-COVID study identified from 1077 post-hospitalization COVID patients four clinical clusters with varying levels of mental and physical impairment between two and seven months post-infection^12^. Depression, anxiety, fatigue, and Post-traumatic Stress Disorder (PTSD) were elevated in two clusters with greatest impairment, whereas cognitive impairment was elevated only in a cluster with moderate impairment^12^. However, K-medioid clustering is limited by its sensitivity to the initial choice of medoids and susceptibility to noise and outliers, which can affect the overall clustering quality. Gaussian mixture models such as latent profile analysis (LPA) share the goal of identifying clinical clusters, but are less sensitive to the initial choice of parameters, noise and outliers, and offer additional advantages in yielding clearer classification boundaries and information on profile assignment^14^. The lack of between-cluster symptom-level comparison may be problematic given the similarity of several depressive, anxiety, and fatigue-related symptoms. Without a contemporaneous non-COVID-infected population control group, it is also difficult to conclude whether COVID-19 infection was associated with increased risk of persistent depression and anxiety.

### The current study

We set out to examine the latent dimensional structure of depression, anxiety, PTSD, fatigue, cognitive, and somatic symptoms in a post-COVID-19 infection cohort (n=248) 12 months post-infection. We included a randomly selected and matched contemporaneous community household sample (n=1834) as a controlled comparison for depressive and anxiety morbidity. The multivariate symptom profiles were further examined on their differentiating features and respective association with functional impairment (Please refer to the Methods section for details).

## Results

### Sample characteristics

All 248 patients had been hospitalized for COVID-19 infection (Until January 2022, all patients infected with COVID-19 in Hong Kong were hospitalised). The sample had similar age distribution (mean = 48.9, SE = 1) and sex ratio (male-46%, female-54%) to those of Hong Kong’s infected population (age mean = 44.6, SE=1; male-to-female ratio 47.5%:52.5%). After weighting, 43.3% received post-secondary education, while 56.9% were married. Average duration since COVID-19 infection was 330 days (47–570 days). 98% in this sample suffered from symptomatic COVID-19 infection, 58.2% of the participants with at least moderately severe infection, similar to the World Health Organization (WHO) and Chinese Centre for Disease Control and Prevention (CCDC) database^15^ (see Supplementary Table S1 online).

### Prevalence of screened current mental, somatic, and cognitive morbidity

One-third of the total sample was screened to have ≥ one mental disorders, PTSD was the commonest (19.1%), followed by Major Depressive Disorder (MDD) (17.4%), Substance Use Disorder (SUD) (13%), and Generalized Anxiety Disorder (GAD) (9.4%). Half of those with a mental disorder reported more than one disorder. 15.9% had moderately severe somatic symptoms. 15.5% reported current suicidal thoughts or behaviours. Compared to the community sample, a significantly higher percentage of COVID-19 survivors screened positive for PTSD (19.1% COVID-19 vs 14% community, p=0.04). Prevalence did not differ for depression, anxiety, alcohol/substance abuse or suicidal thoughts or behaviours (see Supplementary Table S2 online) (Table 1).

**Table 1:** Weighted average symptom score, screened symptom and disorder prevalence (n=248).

	n	%	SE	Mean	SE
Average symptom score					
PHQ9				4.7	0.3
GAD7				3.5	0.3
PCL5				2.9	0.2
PHQ15				5.3	0.4
CFQ				3.5	0.3
AMIC				2.0	0.1
Disorder prevalence					
Depression (PHQ9 ≥ 10)	43	17.4%	2.6%		
Generalized anxiety (GAD7 ≥ 10)	23	9.4%	2.0%		
PTSD (PCL5 ≥ 7)	47	19.1%	2.7%		
Substance/alcohol abuse (CAGEAID ≥1)	32	13.0%	2.3%		
At least 1 disorder	80	32.4%	3.2%		
1 disorder	36	14.6%	2.4%		
2 disorders	25	10.0%	2.1%		
3 disorders	17	6.7%	1.7%		
Symptom prevalence					
Somatic symptoms (PHQ15 ≥ 10)	39	15.9%	2.5%		
Fatigue (CFQ≥ 4)	103	41.5%	3.4%		
SCI (AMIC ≥ 3)	97	38.9%	3.3%		
Suicidal thoughts and behaviours, n (%)	38	15.5%	2.4%		
Co-occurrence of SCI, fatigue and mental disorders					
None	105	42.3%	3.4%		
Only SCI	20	8.2%	1.8%		
Only fatigue	20	7.9%	1.8%		
SCI + fatigue	23	9.2%	1.9%		
Mental disorder	17	6.7%	1.7%		
Mental disorder + SCI	3	1.3%	0.6%		
Mental disorder + fatigue	10	4.2%	1.3%		
Mental disorder + SCI + fatigue	50	20.2%	2.8%		
Logistic regression^1^	OR	95% CI	p		
Any mental disorder ~ SCI and fatigue	8.9	4.5–17.9	< 0.001***		

*PHQ9* patient health questionnaire 9, *GAD7* generalized anxiety disorder 7, *PCL5* posttraumatic stress disorder checklist for DSM-5, *CAGE*-*AID* cut, annoyed, guilty, eye-opener questionnaire adapted to include drugs, *PHQ15* patient health questionnaire 15, *CFQ* Chalder fatigue scale, *AMIC* abbreviated memory inventory for Chinese, *SCI* subjective cognitive impairment.

^1^Logistic regression controlled for past mental disorder and gender.

^***^p<0.001.

41.5% (103) reported significant fatigue (Chalder Fatigue Scale, [CFQ] ≥4). 38.9% (97) reported subjective cognitive impairment (SCI) (Abbreviated Memory Inventory for Chinese [AMIC]>3). Fatigue and SCI overlapped substantially - 51% (127) reported significant fatigue and/or SCI, 29.4% (73) reported both. No data on cognitive impairment or fatigue was available from the community sample for comparison (Table 1).

Mental disorders, fatigue and cognitive impairment overlapped substantially (Table 1). Most with a mental disorder (78.8%) also had SCI or fatigue, whereas 68% of those with fatigue and SCI had a mental disorder. Concurrent SCI and fatigue significantly predicted risk of concurrent mental disorder independent of past history of mental disorder (p<0.001).

### Factors associated with current mental health

Significantly more female (40% vs 24% male) respondents screened positive for at least one mental disorder (X2 (1) = 7.9, p=0.01). Female respondents had 2–3 times increased likelihood to screen positive for depression, anxiety, and suicidal thoughts and behaviour (23% vs 11%; 14% vs 4%; 22% vs 9%). Differences in prevalence of fatigue and SCI were smaller (49% vs 33%; 47% vs 30%), but still significant at p<0.05. Age was negatively correlated with PTSD severity (F[1, 247] = 5.5, p=0.02), but not associated with other disorder or symptom screens (See Supplementary Table S3 online).

29.4% reported a history of mental disorder. History of mental disorder was significantly associated with having MDD, GAD, PTSD, fatigue, SCI (p<0.001), and somatization (p=0.03) (See Supplementary Table S3 online).

Neither COVID-19 infection severity nor time since infection were associated with disorder prevalence (any disorder, MDD, GAD, PTSD, and SUD) nor symptom scores. Mental symptoms or disorders were not more frequent in the 128 respondents (51.6%) with at least moderate COVID-19 infection severity. (see Supplementary Table S4 online) Past mental disorder was not associated with COVID-19 infection severity (p>0.05) (See Supplementary Table S3 online).

### Latent profile analysis (LPA)

**Figure 1:**
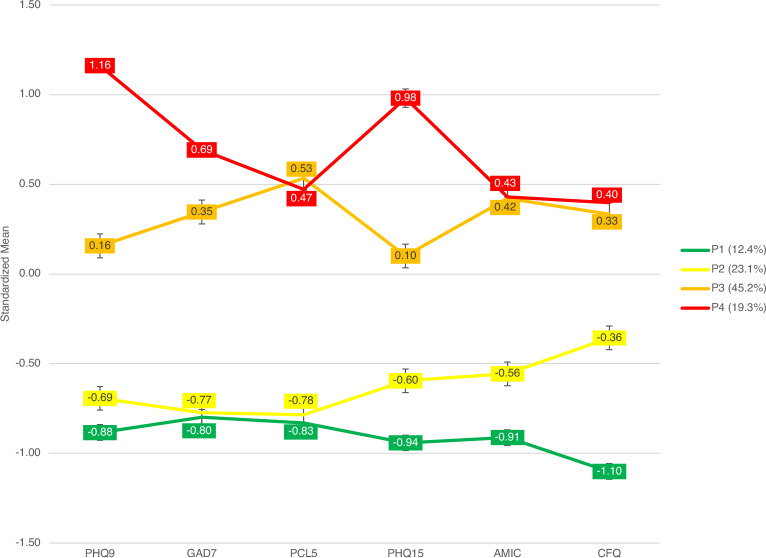
Mean standardized symptom scores across the four latent profiles. Error bars represent 95% confidence interval. *PHQ9* patient health questionnaire 9, *GAD7* generalized anxiety disorder 7, *PCL5* posttraumatic stress disorder checklist for DSM-5, *PHQ15* patient health questionnaire 15, *AMIC* abbreviated memory inventory for Chinese, *CFQ* Chalder fatigue scale.

A 4-component structure (Fig. 1, see Supplementary Table S5 online) provided the best fit for the data based on Bayesian Information Criterion (see Supplementary Table S6 online). Mean classification probabilities for all four profiles were above 95%, suggesting low classification uncertainties from overlapping profile boundaries (see Supplementary Table S7 online).

- Profile one (P1) “No mental morbidity” (12.4% of all participants) had zero mean General Anxiety Disorder-7 (GAD7), Posttraumatic Stress Disorder Checklist for DSM-5 (PCL5), AMIC, and CFQ score and near-zero mean scores for Patient Health Questionnaire 9-item scale (PHQ9), and Patient Health Questionnaire 15 (PHQ15).
- Profile two (P2) “Fatigue and cognitive impairment” (23.1% of all participants) had mildly elevated scores in subjective cognitive impairment (AMIC), fatigue (CFQ), and somatic symptoms (PHQ15), and had similar mental morbidity scores with P1.
- Profile three (P3) “Anxious/PTSD” (45.2% of all participants) constituted the largest group amongst the four profiles, characterized by elevation of all somatic, cognitive, depressive and anxiety symptom scores over P1 and P2.
- Profile four (P4) “Depressive distress” (19.3% of all participants) had further increased in PHQ9, GAD7 and PHQ15 scores compared to P3. Previous mental disorders were more common in those from P3 (37.1%) and P4 (50.7%), compared to 9.9% and 7% in P1 and P2.

Demographic variables (age, sex, education, or marital status) were similar across profiles. Profiles did not differ in COVID-19 infection severity, as measured by hospitalization severity status, days of stay in hospital, drugs applied, or inflammation response (C-reactive protein) (p>0.05) (See Supplementary Table S5 online).

Multivariate analysis of variance (MANOVA) and post-hoc univariate analysis of variance (ANOVAs) found significant between-group differences between the profiles on PHQ9, GAD7, PCL5, PHQ15, CFQ, and AMIC for all six scales (ps<0.001) (Fig. 1, also see Supplementary Table S5 and Supplementary Fig. S1 online).

### Key symptoms differentiating profiles

Using the 51 individual symptom scores from PHQ9, GAD7, PCL5, PHQ15, AMIC, and CFQ, logistic regression models had higher accuracy than random forest models in correctly classifying profile membership in LPA profile pairs. Specifically, the logistic regression models correctly classified 90.5% of LPA profiles in differentiating P2 vs P3, 96.3% for P2 vs P4, and 76.9% for P3 vs P4. In contrast, the random forest models only correctly classified 79.6%, 79.5% and 33.2% of the LPA profiles respectively. P1 was excluded as further train-test splitting was impossible owing to its small group size. To improve interpretability, we applied SHAP (Shapley Additive exPlanations) to the three logistic regression models, which quantifies the contribution of each feature to the prediction, to help identify key symptoms influencing the classifications.

**Figure 2:**
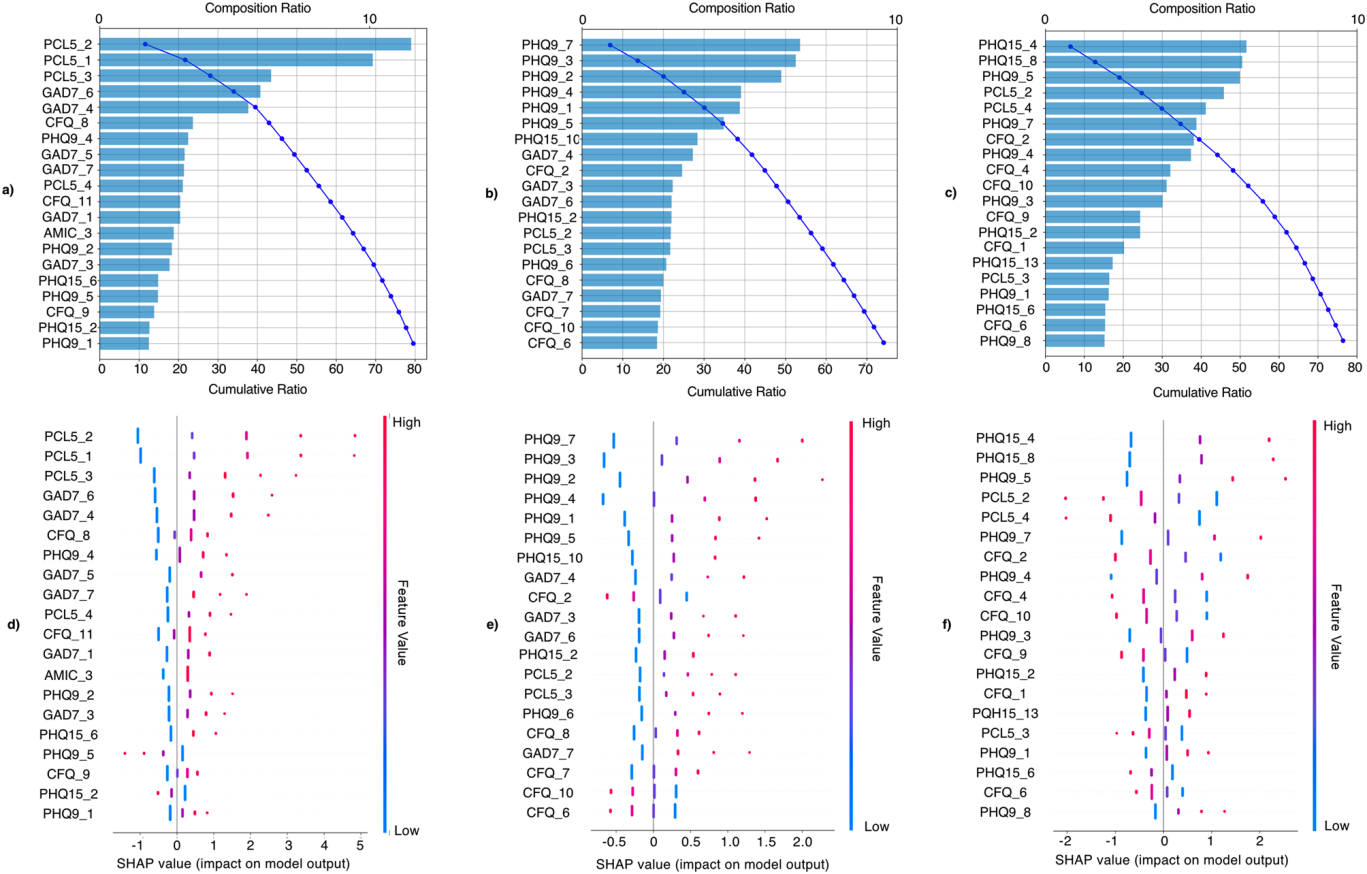
SHAP of symptoms significantly differentiating latent neuropsychiatric and somatic profiles (logistic regression). (**a**–**c**) Waterfall plots of the 20 symptoms with highest SHAP in differentiating p2 vs p3 (**a**); p2 vs p4 (**b**) and p3 vs p4 (**c**). (**d**–**f**) Beeswarm summary plots showing the direction of influence of symptoms in predicting p2 (coded 0) vs p3 (coded 1) (**d**); p2 (0) vs p4 (1) (**e**) and p3 (0) vs p4 (1) (**f**). *PHQ9* patient health questionnaire 9, *GAD7* generalized anxiety disorder 7, *PCL5* posttraumatic stress disorder checklist for DSM-5, *PHQ15* patient health questionnaire 15, *AMIC* abbreviated memory inventory for Chinese, *CFQ* Chalder fatigue scale.

Figure 2 shows the 20 symptoms with the highest SHAP in the logistic regression models in differentiating profile pairs.

Anxiety and PTSD were most important in differentiating P3 from P2, the top five in descending order of importance being *Avoidance*, *Reliving the traumatic event* and *distancing from others* (PCL5), *becoming easily annoyed or irritable*, and *trouble relaxing* (GAD7). The five features each contained >5% of the model’s SHAP, collectively accounting for 39.5% of SHAP. More severe symptoms were associated with a P3 prediction (Fig. 2a,d).

Depressive symptoms were most important in differentiating P4 from P2. The five PHQ9 items with highest SHAP values were *trouble concentrating*, *trouble sleeping*,* feeling down*, *depressed or hopeless*, feeling *tired or having little energy,* and *little interest or pleasure in doing things* (Fig. 2b), and each contained >5% SHAP, collectively accounting for 30% SHAP. More severe symptoms were associated with a P4 prediction (Fig. 2e).

*Headache* and *heart pounding or racing* (PHQ15), *poor appetite* (PHQ9), and *avoidance* and *irritability, angry outbursts or aggressive behavior* (PCL5) were most important in differentiating P4 from P3. The top five symptoms each carried >5%, collectively carrying 29.9% of SHAP. More severe somatic and depressive symptoms were associated with a P4 prediction. More severe PTSD symptoms were associated with a P3 prediction (Fig. 2c,f).

### Psychosocial stressors across LPA profiles

Resilience (Connor-Davidson Resilience Scale [CDRS]) monotonically decreased from P1 to P4 (p<0.001). The profiles did not differ by perceived social support (Oslo Social Support Scale [OSSS-3]). There was significant monotonic increase in reported traumatic experiences in the past year (p=0.04) and negative impact on work-life balance due to COVID-19 (p<0.001) along P1 to P4. Financial stressors were significantly more common in P3 and P4 than P1 and P2. Significant impact on income from COVID-19 (p<0.001) and financial worry (p<0.001) were commonest in P3, followed by P4 (Table 2).

**Table 2:** Stressors across latent profiles (n=248).

	Full sample (N = 248)			P1 (12.4%)			P2 (n = 23.1%)			P3 (n = 45.2%)			P4 (n = 19.3%)					
	n	Mean/%	SE	n	Mean/%	SE	n	Mean/%	SE	n	Mean/%	SE	n	Mean/%	SE	F^a^	DF	Unadjusted p
Past year traumatic event	74	29.8%	3.1%	5	16.7%	7.2%	11	19.7%	5.5%	37	33.4%	4.7%	20	41.9%	7.6%	2.8	(3, 740.64)	0.04*
Knows someone infected	188	75.7%	2.9%	26	84.4%	6.8%	49	84.7%	4.8%	77	68.3%	4.8%	37	76.8%	6.4%	2.2	(3, 739.97)	0.09
Work life balance impacted	90	54.6%	4.3%	3	18.0%	9.9%	9	25.0%	7.7%	52	68.8%	6.0%	25	74.4%	8.5%	9.3	(3, 739.87)	< 0.001***
Finance																		
Household income (HKD)	198	42,083	2204.5	23	38,845	5874.6	46	41,506	3914.5	91.7	43,844	3545.1	37.5	40,461	4919.3	0.2	(3, 187)	0.88
Income significantly impacted	73	29.6%	3.1%	5	16.8%	6.8%	10	17.1%	5.3%	48	42.4%	5.1%	11	22.5%	6.2%	5.1	(2.99, 739.6)	< 0.001***
Financial worry	93	37.4%	3.3%	2	6.6%	4.9%	12	21.7%	5.8%	57	50.5%	5.1%	22	45.4%	7.7%	8.0	(3, 740.61)	< 0.001***
Household																		
Household area (SQFT)	248	481.1	17.8	31	428.6	35.2	57	501.5	50.2	112	475.9	23.6	48	502.6	35.0	0.9	(3, 245)	0.46
Household area)/inhabitants	247	158.8	6.7	31	136.7	11.8	56	170.3	19.3	112	155.7	9.1	48	166.9	12.0	1.3	(3, 244)	0.26
Taking care of child or elderly	121	48.9%	3.4%	12	39.2%	9.3%	33	58.2%	6.7%	54	48.4%	5.1%	22	45.3%	7.7%	1.0	(3, 740.15)	0.39
Physical illnesses																		
Respiratory	21	8.3%	1.7%	1	4.2%	4.1%	8	13.1%	4.5%	7	6.6%	2.1%	4	9.1%	4.2%	0.9	(723.83, 0.43)	0.43
Cardiovascular	27	10.9%	1.8%	3	10.5%	4.7%	7	11.8%	4.0%	13	11.3%	2.7%	4	9.1%	4.2%	0.1	(729.46, 0.96)	0.96
Diabetes	20	8.1%	1.7%	3	9.8%	4.3%	1	2.1%	1.5%	10	9.1%	2.7%	6	12.0%	4.7%	1.7	(710.82, 0.16)	0.16
Cancer	3	1.3%	0.7%	1	4.2%	4.1%	1	1.0%	1.0%	1	1.2%	0.9%	0	0.0%	0.0%	1.0	(661.89, 0.37)	0.37
Chronic liver illness	5	1.9%	1.0%	0	0.0%	0.0%	0	0.0%	0.0%	3	2.4%	1.7%	2	4.2%	3.2%	0.9	(735.22, 0.45)	0.45
Immunological	10	4.1%	1.4%	0	0.0%	0.0%	1	2.5%	2.5%	6	5.4%	2.3%	3	5.5%	3.8%	0.6	(734.46, 0.61)	0.61
Others	56	22.6%	2.8%	6	20.7%	7.2%	10	17.0%	4.9%	30	26.5%	4.5%	10	21.5%	6.4%	0.7	(736.55, 0.57)	0.57
Any of the above	110	44.5%	3.3%	11	37.2%	8.8%	25	42.9%	6.7%	52	45.9%	5.1%	23	47.9%	7.7%	0.3	(739.16, 0.81)	0.81
Social support (OSSS-3)	248	8.3	0.1	31	8.8	0.4	57	8.6	0.3	112	8.1	0.2	48	8.3	0.4	1.3	(3, 245)	0.27
Resilience (CDRS)	248	22.5	0.5	31	28.3	1.1	57	25.9	0.9	112	20.7	0.6	48	19.0	1.1	20.0	(3, 245)	< 0.001***

*OSSS*-3 Oslo social support scale, *CDRS* Connor-Davidson resilience scale.

^a^For categorical variables, this is a variant of the second-order Rao-Scott adjusted chi-square statistic. Significance is based on the adjusted F and its degrees of freedom. For continuous variables, this is a one-way ANOVA.

### Role impairment and LPA profiles

Role impairment (WHO Disability Assessment Schedule [WHODAS]) increased monotonically from P1 to P4 along with increasingly severe mental, cognitive, and somatic symptoms (in P3 and P4) (p <0.001). To explore the independent impact of mental morbidity on role impairment controlled for demographic variables and past mental health, we performed logistic regression with two models (see Supplementary Table S8 online). In Model 1, symptom severity remained significant in predicting impairment independent of past mental health and demographic data, with fatigue as the only symptom dimension among others to predict impairment significantly. Multicollinearity, as detected by variance inflation factor (VIF), was high for depression (PHQ9, VIF=3.77) and anxiety (GAD7, VIF=3.39) in Model 1. In Model 2, latent profiles were found to predict impairment independent of past mental health and demographic data, with P4 significantly predicting impairment. The association of past mental disorders with impairment became insignificant upon multivariate analysis in Model one but remained significant in Model 2 (see Supplementary Table S5 online).

## Discussion

We found that PTSD, depression, anxiety, SCI and fatigue were common and highly comorbid in these 248 consecutively recruited patients one-year post-COVID infection. LPA classified individuals with current neuropsychiatric morbidities (87.6% of the sample) into 3 classes: *P2*–1/4 of the sample, elevated SCI and fatigue only; *P3*–1/3 of the sample, with increased PTSD and anxiety; *P4*–1/5 of the sample with increased depressive features. SCI and fatigue were present in all three groups, but SHAP found anxiety, PTSD and depressive features to be most important in differentiating these classes (P2-4). Classes with elevated anxiety/PTSD and depression (P3 & 4) predicted greatest impairment.

### Comparison with previous prevalence estimates

Both depression and anxiety prevalence estimates in the COVID-19 survivors and community controls were higher than previous Hong Kong community estimates using similar measures^16^, reflecting increased mental distress in the community. Anxiety, depression and PTSD estimates at 1-year follow-up were lower than acute-phase data^17^, consistent with decline with time since infection. Although no other 1-year post-COVID data was available for PTSD, our estimate (19.4%) was similar to usual one-year post-trauma PTSD prevalence figures^18^.

### Dimensional structure of neuropsychiatric and somatic symptoms

PTSD was significantly more common in COVID-19 patients than in community controls, likely related to direct experience of illness. LPA and SHAP revealed the hegemony of PTSD and anxiety –nearly half of the participants were classified into P3, which was distinguished from P4 (depression) and P2 (fatigue/SCI) by anxiety and PTSD features. Seven-month data from the PHOSP-COVID study also yielded a 4-class structure^12^, with increased anxiety, depression, PTSD, and fatigue in the two classes with the greatest impairment. The elevation of psychosocial and dispositional factors in P3, e.g. experience of traumatic event, financial worries, having income significantly impacted by COVID-19, impaired work-life balance, and past mental disorder, further displayed the confluence of factors that may perpetuate anxious/PTSD morbidity^19^.

Previous studies show that fatigue and SCI remained as common one-year post- infection as at 6 months^5^ with substantial overlap with somatization, PTSD, anxiety and depression^20^. LPA and SHAP found fatigue and SCI to be co-ubiquitous across multivariate morbidity classes, but divergent from anxiety, PTSD and depression which were the most important features differentiating P2 (fatigue/SCI), P3 (PTSD/anxiety), and P4 (depression). This confirmed earlier observations of the weak connection of post-COVID-19 cognitive impairment with anxiety/depression^12,13^, providing preliminary support for fatigue and cognitive impairment as core neuropsychiatric features of PCS. The lack of clustering of fatigue with depression or somatization also suggested a divergence of post-COVID-19 fatigue from somatization^21^, echoing findings that anxiety/depression did not predict post-COVID-19 fatigue^22^.

### Mental health and COVID-19 severity

COVID-19 infection has been proposed to affect mental health by neuroinflammatory mechanisms^10^, but the association between infection severity and subsequent depressive and anxiety symptoms has not been consistently observed^12,23^. Unlike the 6-month cognitive test data from 46 COVID patients in the Addenbrookes study^13^, we did not find symptom/profile-level association with acute infection and treatment-related markers. This could result from differences in sample size, smaller number of severely infected cases, attenuation of biological effects from infection and treatment 12 months from infection^24^.

#### LPA profiles and impairment

Unlike disorder-based logistic regressions, logistic regression with LPA profiles had no significant multicollinearity and found past mental health and P4 to significantly predict impairment, consistent with the common association of chronic mental conditions with health outcomes and depression with greater impairment compared with anxiety. These results should be examined in larger samples, but highlight the salience of mental health care in this substantial population with PTSD, anxiety and depression after COVID-19^6^.

### Strengths and limitations

Strengths of this study included a sample demographically similar to the local COVID-19 population, matched comparison with a randomly selected non-COVID infected household, use of validated screening instruments, and the multi-variate dimensional insight offered by LPA and SHAP analysis. The limitations below should however be considered.

First, the generalisability of the findings is heavily limited by both the low response rate (39.3%) and the single-region sample. Regarding the low response rate Although non-responders did not differ in age or gender from responders, we were unfortunately unable to access clinical data of non-responders as most had not provided consent to participate in the study. Without a comparison of the clinical characteristics of responders versus non-responders, we cannot assess the level of selection bias in terms of clinical severity and heterogeneity that would have likely resulted from the low response rate. The fact that the study was conducted in a single region also limits the generalizability of the results to other populations and settings. Future efforts to including data from this study in multi-national/regional analyses with similar multivariate approaches would be salient to examining the external validity of the findings here. Second, we did not prospectively collect concurrent psycho-behavioural and biological data in the acute/pre-infection phases. The chronological association between mental health and the biological and neuropsychiatric manifestations of COVID-19 infection^3,9^ will hopefully be clarified in longitudinal follow-up of this sample of COVID and non-infected community individuals. Third, our small sample size may affect validity of the LPA profiles, which should be considered preliminary and be examined in larger samples. Also, we did not include non-hospitalized patients, as all positively tested individuals in Hong Kong were hospitalized until the 2022 COVID-19 surge made this impossible^5,23^. Ongoing data collection will provide useful insights as many infected individuals in our prospective sample were no longer hospitalised. Fourth, diagnosis of mental disorders could not be made in the absence of clinical interviews. Self-rated questionnaires, although validated and psychometrically robust, may inflate prevalence estimates. AMIC accurately predicted objectively measured cognitive impairment and identified individuals with major neurocognitive disorder, but was not a cognitive test^25^^,^^13^. Fifth, we did not measure cognitive impairment, fatigue, or somatisation in the community sample, which was originally intended for estimating the pandemic’s population psychological impact in 2020 when infection rate in Hong Kong was low. With the tremendous spread of COVID-19 in Hong Kong in 2022, we will also measure fatigue, somatisation, and include online web-based psychological tests in follow-up assessments for both infected and non-infected community participants, to yield fine grained data with superior time resolution^26^. Sixth, PHQ15 measured somatic symptoms, but was not a COVID-specific symptom measure. Interestingly, the PHOSP-COVID study found dyspnoea and physical performance to cluster with anxiety and depression, similar to the PHQ15 items in our study^12^. Additionally, natural groupings corresponding to LPA profiles may not exist, as the profiles were only best-fits identified relative to each other. Although the > 95% average prediction probabilities in our four profiles made this unlikely, arbitrary profile assignment and unreliable estimates of group properties may occur where profiles are poorly separated. Finally, the statistical power of SHAP can be affected by differences in the questions’ response structure. For example, AMIC as a binary measure may yield lower statistical power than Likert-based measures.

We presented initial evidence to support cognitive impairment and fatigue as core features of PCS/long-COVID. Increased risk of PTSD, high prevalence of anxiety and depression, and associations of these profiles with environmental risk factors and functional impairment highlighted the psychosocial determinants of health outcomes in infectious diseases^27^. Prospective multi-modality examination of larger samples should verify these preliminary results, but if these estimates of mental morbidity and impairment hold true for the massive populations affected by COVID-19, they would attest to a ‘mental health pandemic’ with serious public health implications. Web-based psychological interventions with population-level implementation potential^28^ are now being tested in the CU-COVID-19 study for their effectiveness in alleviating anxiety and depressive distress.

## Methods

### Study design

This paper reports the baseline survey results of 248 COVID-19 survivor participants in the CU-COVID19 study, a multi-centre web-based observational cohort study of the incident mental morbidity of COVID-19 survivors, healthcare workers, ex-quarantine confines, and the general Hong Kong population.

This analysis included all 248 baseline surveys that were completed between March-2021 to January-2022 by individuals who were hospitalized for COVID-19 on average 321 days (47–570 days) since hospitalization. *(All COVID-19 cases in Hong Kong were hospitalized up to January 2022.)* Respondents were recruited from a Chinese University of Hong Kong (CUHK) research database consisting of 751 COVID-19 patients with verified hospital admissions and clinical data. Patients in the database were considered eligible if they were aged 18-75 and able to read Chinese. 631 patients were eligible after excluding 19 patients aged over 75 and 101 patients unable to read Chinese.

To minimise response bias, we had systematically reached all 631 eligible COVID-19 patients 3 times each. Of the eligible patients, 158 declined and 109 were unreachable. The remaining 364 patients had all provided informed consent to participate in the study and received a confidential Qualtrics survey link via email or text messages. To maximise completion, shopping coupons worth HKD100 were offered as incentives, and up to 5 biweekly SMS or emails were sent to those with an incomplete response. Of the 364 consented participants, 248 completed the survey (Please see Supplementary Fig. S2 online for a recruitment flowchart). Response rate was 39.3% (248 completed/ 631 eligible). There were no significance differences in the age and gender of responders and non-responders (Age T (629) =0.068, p=0.95; Gender X^2^ (1) = 1.04 p=0.3). We were not able to access clinical data of non-responders as most had not provided consent to this study.

The authors assert that all procedures contributing to this work comply with the ethical standards of the relevant national and institutional committees on human experimentation and with the Helsinki Declaration of 1975, as revised in 2008. All procedures involving human subjects/patients were approved by the Clinical Research Ethics Committee of the Hong Kong New Territories East cluster, The Hong Kong Island East and Kowloon West cluster (Ref no. 2020.338-T).

### Assessments

The COVID-19 Mental Health impact survey was developed and standardized in collaboration with the World Mental Health Survey COVID workgroup^29,30^, including the following items:

Socio-demographic data, neuropsychiatric symptoms, and somatic symptoms were assessed at the same time during the survey, which was completed between March-2021 and January-2022.

Socio-demographic data included age, sex, education, and marital status. Depression was screened using the full Chinese PHQ9 which included one item on fatigue, with a cut-off score of ≥10. The Chinese PHQ9 had demonstrated good psychometric properties, including an internal consistency reliability of 0.86 and a 2-week test-retest correlation of 0.86^31^. Anxiety was assessed with the Chinese GAD7 with a cut-off score of ≥10, which showed a Cronbach’s alpha of 0.91 and an AUC of 0.88 for discriminating GAD^32^. SUD was determined using the Chinese CAGE-AID Questionnaire (“Cut, Annoyed, Guilty, Eye-opener” questionnaire Adapted to Include Drugs) with a cut-off score of ≥1, which exhibited an AUC of 0.7, sensitivity of 66.7%, and specificity of 67.5%^33^. PTSD was assessed using the PCL-5. The 20-item Chinese PCL-5 had demonstrated good psychometric properties, with a Cronbach’s Alpha of 0.91 as well as good convergent and discriminant validity^34^. The 20-item PCL-5 had been shortened into a 4-item version for brevity. The 4-item English PCL-5 had good internal consistency (0.82) and accounted for 87% of the variance in the 20-item PCL5. In our Chinese sample, we found a Cronbach’s alpha of 0.876 for the 4-item PCL5^35^.

Thirty-day suicidal thoughts and behaviours was screened as any self-report of self-harm, suicidal thought, ideation, plan or action in the past 30 days, at least one month in the past year or considered likely to act on these thoughts in the next 3 months.

Somatic symptoms and fatigue were screened using the PHQ15 with a cut-off score of ≥10 and the CFQ with a cut-off score of ≥ 4. The Chinese version of the PHQ15 demonstrated good reliability with a Cronbach’s alpha of 0.83 and discriminant validity from the PHQ9 and GAD7^34^. The Chinese version of the CFQ showed a Cronbach’s alpha of 0.86, good convergent validity with the Hospital Anxiety and Depression Scale, and divergent validity with the 12-Item Short Form Survey^36^. SCI was rated by the 5-item AMIC^25^, with cut-off point ≥3. Items included self-reports of ‘forgetting where things are placed’, ‘unable to recall the names of good friends’, ‘unable to follow and recall conversation’, ‘subjective memory problems’, and ‘memory to be worse than others of a similar age’. The sensitivities of AMIC in identifying mild cognitive impairment possible incipient dementia and early dementia are 65.3% and 70.4% respectively.

Role impairment was measured using a simplified Work Loss Days index from the validated version of the WHODAS 2.0, which demonstrated a Cronbach’s alpha of 0.85–0.98 across 16 countries^37^. Social support was assessed with the OSSS-3, which had an acceptable internal consistency with a Cronbach’s alpha of 0.64 and a one-factor solution validated in confirmatory factor analysis^38^. Resilience was measured using the CDRS, with the Chinese version exhibiting a Cronbach’s alpha of 0.877 and a test-retest correlation of 0.73^39^.

Participants indicated lifetime mental disorders from a list (depression, bipolar disorder, panic attacks, anxiety related problems, alcohol or SUD) or a territory-wide public hospital electronic registry showing record of diagnosis, treatment or prescription for any mental health problems before the COVID-19 outbreak in January 2020. Clinical and laboratory data on COVID-19 infection severity and treatment was retrieved from hospital electronic records with explicit consent from participants.

Participants also indicated whether they had in the past year experienced traumatic events, knew someone who was infected, experienced financial stress, reduced household income from the pandemic, or had physical illnesses apart from COVID-19.

### Statistical analysis

Unless otherwise specified, all analyses were age-sex weighted to Hong Kong’s COVID-19 infected population (n=10,830 circa 18 December 2021).

Demographics, COVID-19 severity and treatment, prevalence of mental disorders, somatization, fatigue, and SCI were reported. The effects of sex and age on symptom severity (average symptom scores) and disorder prevalence were examined via chi-square and logistic regression. The prevalence of positive screens and average scores for depression, anxiety, PTSD, and SUD in COVID-19 survivors were compared against a community sample (n=1834, representative household sample collected via mailing survey invitations to 10,000 randomly selected addresses generated by the Census Department between Nov-20 and Mar-22) age-sex weighted to the local COVID-19 population. Association of time since infection with disorder prevalence and symptom severity were tested using logistic and linear regression. Infection severity, mental disorder prevalence and symptom scores were compared between respondents with versus without a history of mental disorder using t-tests and chi-square tests.

We employed LPA to explicate the latent relationships between cognitive impairment, fatigue and neuropsychiatric symptoms. LPA was performed using Mclust (v.5.4.8) in R, with PHQ9, GAD7, PCL5, PHQ15, CFQ, and AMIC in the data matrix. The Mclust package models data as a Gaussian finite mixture under different covariance structures and number of mixture components. Bayesian Information Criterion (BIC) was selected a priori as criterion for parameter optimization.

Multivariate profiles generated by LPA were compared on demographics, COVID-19 infection severity and treatment, mental symptom scores, past mental disorders, treatment history, stressors experienced during the pandemic, social support, resilience, and impairment using chi-square/univariate ANOVA tests. Between-group differences were tested using unweighted MANOVA, with univariate Sidak correction for multiple comparison for each of the six scales.

To identify the most important symptoms distinguishing the profiles, 51 unweighted individual symptom scores from PHQ9, GAD7, PCL5, PHQ15, AMIC, and CFQ were used to build machine learning classifiers (logistic regression and random forest) to differentiate profile pairs. Data was split into training and tests sets with a 75:25 ratio, stratified on profile labels.

Model optimization was performed on the training set using grid search, with available options including number of estimators 10–300 in steps of 10, maximum number of features ‘auto’ or ‘sqrt’, maximum depth between 10 and 110 in steps of 10, minimum samples split and minimum sample leaf 1–10, with or without bootstrap.

The optimized models were evaluated based on their ability to correctly predict LPA labels based on the 51 symptom scores in the unseen test set. Specifically, we defined accuracy as the proportion of correctly classified labels in the test set by the optimized model.

An interpretation algorithm, SHAP (Python SHAP v0·4), was then applied to the most accurate model to assess symptom importance. SHAP assigns to each feature an importance value based on how much it pushes the predicted outcome from a base value. SHAP values improve the interpretability of machine learning models by showing the direction and ratio of each feature’s contribution to a model without reference to the original scale^40^.

To validate the profiles, comparison was made of linear regression models of impairment (WHODAS) on demographic variables, history of mental disorder, and average symptom scores (Model 1) versus latent profile grouping (Model 2). Standardized beta estimates for each predictor were presented as part of univariate, and multivariate regression controlling for other predictors. Variance inflation factors assessed multicollinearity with each variable. The contributions of newly included variables were tested stepwise using F-tests. The two models were evaluated individually using R-squared, adjusted r-squared, and *F*-test (compared to intercept only).

Latent class analysis and WHODAS linear models were performed using R v.4.1.2. SHAP. Random forest and logistic regression models were run on Python v 3·7·4. The remaining analyses were performed using SPSS v25. A Sidak corrected alpha of 0·0085 (1–[1–0·06]^[1/6]) was used in the post-hoc univariate ANOVAS comparing the 6 scales in the LPA groups. For other tests, an alpha level of 0·05 was used. T-tests were two-tailed. All other tests were one-tailed.

The R and Python code used in this analysis are available at a public repository at https://github.com/owennwl/CUCOVID19_DXLPA.

## Supplementary Information

Supplementary Information.

Supplementary Figure S1.

Supplementary Figure S2.

## Data Availability

Requests for data should be directed to the corresponding author [Mak, Arthur D.P.]. Data will be available upon reasonable request.
