# Supplementary material for: Dimensional structure of one-year post-COVID-19 neuropsychiatric and somatic sequelae and association with role impairment
Source: Sci Rep. 2023 Jul 27;13:12205. doi: 10.1038/s41598-023-39209-z (PMC10374659; doi:10.1038/s41598-023-39209-z)
Supplement: Supplementary file 1 — Supplementary Information. [file 41598_2023_39209_MOESM1_ESM.pdf]

Supplementary Material

Dimensional Structure of one-year Post-COVID-19 Neuropsychiatric and Somatic Sequelae and Association with Role Impairment

Owen N.W. Leung[a], BSocSc[#]; Nicholas K.H. Chiu[a], MBChB[#], Samuel Y.S. Wong[b], MD; Pim Cuijpers[c, d], PhD; Jordi Alonso[e], PhD; Paul K.S. Chan[g], MD; Grace Lui[h], MRCP; Eliza Wong[b], PhD; Ronny Bruffaerts[i], PhD; Benjamin H.K. Yip[b], PhD; Philippe Mortier[f], PhD; Gemma Vilagut[f], PhD; Dora Kwok[g], MA; Linda C.W. Lam[a], MD; Ronald C. Kessler[j], PhD; Arthur D.P. Mak[a], FRCPsych[*]

[a]Department of Psychiatry, Faculty of Medicine, The Chinese University of Hong Kong, HK

[b]Jockey Club School of Public Health and Primary Care, The Chinese University of Hong Kong, HK

[c]Department of Clinical, Neuro and Developmental Psychology, Amsterdam Public Health Research Institute, Vrije Universiteit Amsterdam, Amsterdam, The Netherlands

[d]Babeș-Bolyai University, International Institute for Psychotherapy, Cluj-Napoca, Romania

[e]Health Services Research group, IMIM-Institut Hospital Mar d'Investigacions Mèdiques, Universitat Pompeu Fabra, CIBERESP, Barcelona, Spain

[f]Health Services Research group, IMIM-Institut Hospital Mar d'Investigacions Mèdiques, CIBER Epidemiología y Salud Pública CIBERESP Barcelona ES

[g]Department of Microbiology, Faculty of Medicine, The Chinese University of Hong Kong, HK

[h]Department of Medicine and Therapeutics, Faculty of Medicine, The Chinese University of Hong Kong, HK

[i]Universitair Psychiatrisch Centrum - Katholieke Universiteit Leuven (UPC-KUL), Campus Gasthuisberg, Leuven, Belgium

[j]Department of Health Care Policy, Harvard Medical School, Boston, Massachusetts, USA

* Corresponding author
Dr. Arthur Dun-Ping Mak
Department of Psychiatry, The Chinese University of Hong Kong
G/F Multicentre, Tai Po Hospital, Tai Po, Hong Kong SAR
arthurdpmak@cuhk.edu.hk
Tel (852) 2831 4305 Fax: (852) 2447 6669
\# Joint first authors.

Supplementary Table S1. Weighted demographics, treatment and acute COVID19 illness severity (N=248)

	n	%	SE	Mean	SE
**Demographics**					
Age					
Sample (Before weighting)				48.9	1.0
Population (After weighting)				44.6	1.0
Sex, male					
Sample (Before weighting)	114	46.0%	3.20%		
Population (After weighting)	118	47.5%	3.40%		
Education					
Primary or below	20	8.1%	1.50%		
Secondary	120	48.6%	3.40%		
Post-secondary	107	43.3%	3.40%		
Marital status					
Single	87	35.3%	3.30%		
Married	141	56.9%	3.40%		
Divorced	11	4.3%	1.30%		
Widowed	9	3.5%	1.10%		
	**n**	**%**	**SE**	**Mean**	**Range (Q1-Q3)**
**Treatment**					
Days in hospital				17.2	2 - 114 (10-19)
Days since admission				329.9	47 - 570 (209-409)
Intensive care unit stay	14	6%	1.7%		
Supplemental oxygen	30	14%	2.3%		
Mechanical ventilation	8	4%	1.3%		
Drugs					
Lopinavir	72	32%	3.4%		
Ribavrin	84	37%	3.4%		
IFN	106	47%	3.6%		
Remdesivir	27	12%	2.1%		
Steroid	33	15%	2.4%		
	**n**	**%**	**SE**	**Mean**	**SE**
**Acute COVID19 severity**					
C reactive protein (mg/L)				1.6	0.2
Severity[a]					
Asymptomatic	4	2%	1.0%		
Mild	87	40%	3.6%		
Moderate	100	45%	3.6%		
Severe	15	7%	1.6%		
Critical	14	7%	1.7%		
Neuro symptom	73	48%	4.3%		
Cardiac symptom	12	9%	2.5%		
Viral pneumonitis	128	58%	3.6%		
Secondary infections	11	5%	1.5%		

[a]Mild = No pneumonia but with other sypmtoms; Moderate = With pneumonia; Severe = Pneumonia with desaturation requiring O_2; Critical = Mechanical ventilation shock or ICU

Supplementary Table S2. Comparison of symptom scores and prevalence between COVID-19 survivors and community respondents

	Community (n=1834)[a]		COVID-19 survivors (n=248)				
	Mean	SE	Mean	SE	t	df	p
**Symptom score**							
PHQ9	4.3	0.1	4.7	0.3	0.9	2080	0.36
GAD7	3.6	0.1	3.5	0.3	-0.4	2080	0.70
PCL5	2.9	0.1	2.9	0.2	-0.1	2080	0.94
CAGEAID	0.2	0.0	0.2	0.0	0.8	2080	0.40
	N	%	n	%	X^2	df	p
**Disorder/ symptom prevalence**							
Depression (PHQ9 >=10)	258	14.1%	43	17.4%	1.9	1	0.20
Generalized anxiety (GAD7 >=10)	190	10.4%	23	9.4%	0.2	1	0.68
PTSD (PCL5 >=7)	257	14%	47	19.1%	4.5	1	0.047*
Substance/alcohol abuse (CAGEAID >=1)	166	9.1%	32	13%	3.9	1	0.06
Any disorder	497	27.1%	80	32.4%	3.1	1	0.1
Suicidal thoughts or behaviours	329	18%	38	15.5%	0.9	1	0.37

[a] Representative community sample via mailing survey invitations to 10,000 randomly drawn addresses. Surveys were completed between Nov 20 and Mar 22.
Both the community and COVID-19 survivors samples were weighted to the age-sex distribution of all COVID-19 survivors recorded on government database as of 18-Dec-21
* = p<.05

Supplementary Table S3. Effects of sex, age, COVID-19 infection severity, time since admission and previous mental disorders on disorder/symptom prevalence and severity (N=248)

	Sex			Age			COVID-19 infection severity (at least moderate) [b]			Time since admission			Previous mental disorder		
	X^2	df	p	F [a]	df	p	$\chi2$	df	p	F [a]	df	p	$\chi2$	df	p
**Symptom prevalence**															
Depression (PHQ9 >=10)	5.8	1	0.03*	0.8	(1, 247)	0.37	0.1	1	0.82	0.0	(1, 222)	0.97	27.8	1	<0.0001***
Generalized anxiety (GAD7 >=10)	7.9	1	0.01*	1.1	(1, 247)	0.30	0.1	1	0.81	0.0	(1, 222)	1.00	20.0	1	<0.0001***
PTSD (PCL5 >=7)	3.3	1	0.1	2.0	(1, 247)	0.16	0.7	1	0.44	1.0	(1, 222)	0.31	20.0	1	<0.0001***
Substance/alcohol abuse (CAGEAID >=1)	3.8	1	0.06	0.0	(1, 247)	0.89	0.2	1	0.65	0.3	(1, 222)	0.59	0.7	1	0.42
Any disorder	7.9	1	0.01*	0.3	(1, 247)	0.59	0.6	1	0.45	0.1	(1, 222)	0.77	23.6	1	<0.0001***
Somatic symptoms (PHQ15 >=10)	2.1	1	0.18	0.2	(1, 247)	0.69	2.6	1	0.12	1.0	(1, 222)	0.33	5.2	1	0.03*
Fatigue (CFQ >=4)	6.7	1	0.02*	0.4	(1, 247)	0.51	0.9	1	0.38	0.0	(1, 222)	0.94	16.5	1	<0.0001***
SCI (AMIC >=3)	7.7	1	0.01*	2.7	(1, 247)	0.10	0.8	1	0.42	0.6	(1, 222)	0.45	16.1	1	<0.0001***
Suicidal thoughts or behaviours	7.8	1	0.01*	2.3	(1, 247)	0.14	0.3	1	0.61	2.0	(1, 222)	0.16	6.9	1	0.01*
**Symptom score**	**t**	**df**	**p**	**F**	**df**	**p**	**t**	**df**	**p**	**F**	**df**	**p**	**t**	**df**	**p**
PHQ9	2.4	247	0.02*	0.4	(1, 247)	0.52	0.9	218	0.36	0.8	(1, 222)	0.39	-5.9	247	<0.0001***
GAD7	2.9	247	0.004**	2.3	(1, 247)	0.13	2.0	218	0.05	1.3	(1, 222)	0.25	-5.9	247	<0.0001***
PCL5	2.8	247	0.006**	5.5	(1, 247)	0.02*	1.1	218	0.27	2.8	(1, 222)	0.10	-5.7	247	<0.0001***
PHQ15	1.4	247	0.17	0.2	(1, 247)	0.69	1.8	218	0.08	2.1	(1, 222)	0.15	-3.4	247	0.00039***
CFQ	2.5	247	0.01*	1.6	(1, 247)	0.21	1.1	218	0.26	1.3	(1, 222)	0.26	-5.2	247	<0.0001***
AMIC	2.4	247	0.02*	0.7	(1, 247)	0.40	0.9	218	0.38	2.5	(1, 222)	0.12	-4.1	247	<0.0001***

[a]Wald test
[b]With pneumonia, requiring mechanical ventilation shock or admission to ICU
PHQ9, Patient Health Questionnaire 9; GAD7, Generalized Anxiety Disorder 7; PCL5, Posttraumatic Stress Disorder Checklist for DSM-5; CAGE-AID, Cut, annoyed, guilty, eye-opener questionnaire adapted to include drugs; PHQ15, Patient Health Questionnaire 15; CFQ, Chalder Fatigue Scale; AMIC, Abbreviated Memory Inventory for Chinese; SCI, subjective cognitive impairment
p values were not adjusted for multiple comparison
*, **, *** = p<.05, .01, .001

Supplementary Table S4. Effect of past mental disorder on COVID-19 severity (n=248)

	No (70.6)			Yes (29.4)					
	n	%	SE	n	%	SE	$\chi2$	DF	unadjusted p
**COVID-19 severity[a]**							4.1	4	0.44
Asymptomatic	4	2.8%	1.5%	0	0%	0%			
Mild	59	38.5%	4.2%	28	42.4%	6.7%			
Moderate	71	46.3%	4.3%	28	42.7%	6.6%			
Severe	8	5.2%	1.7%	7	10.0%	3.7%			
Critical	11	7.2%	2.1%	3	4.9%	2.7%			

[a]Mild = No pneumonia but with other sypmtoms; Moderate = With pneumonia; Severe = Pneumonia with desaturation requiring O_2; Critical = Mechanical ventilation shock or ICU

Supplementary Table S5. Demographics, past mental health problems, baseline COVID19 severity, current mental health symptoms and role impairment across 4 latent profiles (n=248)

	P1 (12.4%)			P2 (23.1%)			P3 (45.2%)			P4 (19.3%)					
	**n**	**Mean/ %**	**SE**	**n**	**Mean/ %**	**SE**	**n**	**Mean/ %**	**SE**	**n**	**Mean/ %**	**SE**	**F[a]**	**DF**	**unadjusted p**
**Demographics**															
Age	31	47.2	3.1	57	47.0	2.1	112	42.3	1.4	48	45.5	2.1	1.7	(3, 245)	0.18
Sex, male	18	58.5%	9.3%	30	51.9%	6.9%	51	45.8%	5.1%	19	39.5%	7.7%	1.0	(3, 740)	0.41
Education													1.0	(6, 1394)	0.39
Primary or below	2	7.6%	3.8%	7	11.5%	3.9%	8	6.8%	2.1%	4	7.8%	3.2%			
Secondary	19	60.7%	9.4%	27	47.2%	6.9%	48	42.9%	5.0%	27	55.7%	7.7%			
Post-secondary	10	31.7%	9.3%	24	41.3%	6.8%	56	50.3%	5.1%	18	36.6%	7.7%			
Marital status													1.7	(8, 2074)	0.10
Single	8	27.2%	9.2%	15	25.8%	6.2%	47	41.8%	5.1%	17	36.4%	7.6%			
Married	22	70.9%	9.2%	39	68.1%	6.4%	58	51.5%	5.1%	23	47.2%	7.7%			
Divorced	1	1.9%	1.9%	2	3.1%	1.8%	5	4.3%	1.9%	3	7.2%	4.3%			
Widowed	0	0.0%	0.0%	2	3.0%	1.7%	3	2.3%	1.5%	4	9.2%	4.0%			
**Past mental disorder**	3	9.9%	6.5%	4	7.0%	3.6%	42	37.1%	4.9%	24	50.7%	7.8%	8.7	(2, 732)	0.00018***
**Mental health symptoms**															
PHQ9	31	0.2	0.1	57	1.1	0.2	112	5.3	0.4	48	10.3	0.8	68.6	(3, 244)	<0.0001***
GAD7	31	0.0	0.0	57	0.1	0.0	112	4.9	0.4	48	6.4	0.7	51.4	(3, 244)	<0.0001***
PCL5	31	0.0	0.0	57	0.1	0.0	112	4.4	0.3	48	4.2	0.5	52.3	(3, 244)	<0.0001***
PHQ15	31	0.7	0.1	57	2.4	0.3	112	5.9	0.4	48	10.4	1.0	49.0	(3, 244)	<0.0001***
CFQ	31	0.0	0.0	57	1.4	0.2	112	2.6	0.2	48	2.8	0.3	34.4	(3, 244)	<0.0001***
AMIC	31	0.0	0.0	57	1.3	0.2	112	4.9	0.4	48	5.0	0.7	29.4	(3, 244)	<0.0001***
**Baseline COVID19 severity**															
Days in hospital	30	15.0	1.9	49	15.6	1.2	105	19.0	1.4	39	16.1	1.8	1.4	(3, 220)	0.26
Severity[b]													1.1	(11, 2791)	0.40
Asymptomatic	1	2.7%	2.7%	1	2.9%	2.9%	0	0.0%	0.0%	2	5.4%	4.0%			
Mild	17	55.2%	9.3%	20	41.0%	7.6%	37	35.7%	5.1%	14	36.4%	8.6%			
Moderate	9	29.7%	7.9%	25	50.7%	7.6%	50	48.3%	5.3%	16	42.2%	8.5%			
Severe	2	5.8%	3.4%	1	1.2%	1.2%	9	8.8%	2.8%	3	8.4%	4.4%			
Critical	2	6.6%	4.9%	2	4.1%	2.4%	7	7.2%	2.5%	3	7.6%	5.1%			
Intensive care unit stay	2	6.6%	4.9%	2	4.1%	2.4%	7	7.1%	2.4%	3	7.3%	4.9%	0.2	(3, 705)	0.90
Supplemental oxygen	4	12.4%	5.8%	3	5.3%	2.7%	17	15.8%	3.6%	7	19.0%	6.9%	1.5	(3, 715)	0.20
Neuro symptom	4	25.5%	11.6%	17	49.8%	9.1%	35	47.1%	6.2%	17	61.4%	9.8%	1.6	(3, 741)	0.20
Cardiac symptom	1	4.0%	4.0%	5	15.9%	6.6%	6	9.4%	3.9%	0	0.0%	0.0%	1.7	(3, 693)	0.17
Viral pneumonitis	13	42.1%	9.1%	27	56.0%	7.6%	66	64.3%	5.1%	22	58.2%	8.7%	1.4	(3, 739)	0.23
Secondary infections	3	9.5%	6.3%	1	2.9%	2.1%	4	4.0%	1.8%	2	5.1%	3.8%	0.6	(3, 711)	0.58
Mechanical ventilation	1	4.7%	4.6%	1	2.9%	2.1%	4	4.0%	1.8%	1	3.6%	3.5%	0.1	(3, 703)	0.98
C reactive protein (mg/L)	29	2.0	0.6	47	1.6	0.3	105	1.7	0.3	39	1.3	0.3	0.5	(3, 216)	0.69
Drugs															
Lopinavir	7	24.8%	8.9%	17	34.0%	7.0%	39	37.4%	5.1%	8	20.2%	7.1%	1.3	(3, 739)	0.27
Ribavrin	10	34.1%	9.1%	20	41.0%	7.2%	42	39.8%	5.1%	11	28.8%	8.0%	0.6	(3, 740)	0.64
IFN	13	43.3%	9.5%	22	45.0%	7.4%	57	54.8%	5.3%	13	34.2%	8.2%	1.6	(3, 740)	0.19
Remdesivir	4	14.4%	6.1%	5	9.4%	4.0%	16	14.8%	3.5%	2	6.1%	3.0%	1.0	(3, 725)	0.39
Steroid	5	16.6%	6.4%	3	5.2%	2.6%	20	18.7%	3.8%	6	15.4%	6.3%	1.8	(3, 715)	0.15

Supplementary Table S5. Demographics, past mental health problems, baseline COVID19 severity, current mental health symptoms and role impairment across 4 latent profiles (n=248)

	P1 (12.4%)			P2 (23.1%)			P3 (45.2%)			P4 (19.3%)					
	**n**	**Mean/ %**	**SE**	**n**	**Mean/ %**	**SE**	**n**	**Mean/ %**	**SE**	**n**	**Mean/ %**	**SE**	**F[a]**	**DF**	**unadjusted p**
**Role impairment**															
WHODAS	31	7.2	4.8	57	8.8	2.8	112	20.1	2.6	48	27.8	4.4	6.5	(3, 245)	0.0003***

PHQ9, Patient Health Questionnaire 9; GAD7, Generalized Anxiety Disorder 7; PCL5, Posttraumatic Stress Disorder Checklist for DSM-5; CAGE-AID, Cut, annoyed, guilty, eye-opener questionnaire adapted to include drugs; PHQ15, Patient Health Questionnaire 15; CFQ, Chalder Fatigue Scale; AMIC, Abbreviated Memory Inventory for Chinese; SCI, subjective cognitive impairment

[a]For categorical variables, this is a variant of the second-order Rao-Scott adjusted chi-square statistic. Significance is based on the adjusted F and its degrees of freedom. For continuous variables, this is a one-way ANOVA.

[b]Mild = No pneumonia but with other sypmtoms; Moderate = With pneumonia; Severe = Pneumonia with desaturation requiring O_2; Critical = Mechanical ventilation shock or ICU

*** = p<.001

Supplementary Table S6. Bayesian Information Criterion for different covariance structures and number of components

		Bayesian Information Criterion													
		EII	VII	EEI	VEI	EVI	VVI	EEE	VEE	EVE	VVE	EEV	VEV	EVV	VVV
Number of profiles	1	-4255.3	-4255.3	-4282.9	-4282.9	-4282.9	-4282.9	-3429.3	-3429.3	-3429.3	-3429.3	-3429.3	-3429.3	-3429.3	-3429.3
	2	-3574.6	-3249.5	-3575.8	-3268.6	NA	NA	-3468.1	-2832.2	NA	NA	-3368.7	-2630.5	NA	NA
	3	-3457.1	-2817.5	-3434.3	-2787.1	NA	NA	-3313.9	-2700.9	NA	NA	-3372.4	-2535.5	NA	NA
	4	-3495.7	-2716.6	-3409.5	-2629.1	NA	NA	-3409.6	-2612.6	NA	NA	-3356.0	-2349.34*	NA	NA
	5	-3452.9	-2695.6	-3400.2	-2585.2	NA	NA	-3367.4	-2591.4	NA	NA	-3346.8	-2461.1	NA	NA
	6	-3457.4	NA	-3402.0	-2521.3	NA	NA	-3372.6	-2595.1	NA	NA	-3367.4	-2531.4	NA	NA
	7	-3338.1	NA	-3312.9	-2491.0	NA	NA	-3364.9	-2591.0	NA	NA	-3419.3	-2504.8	NA	NA
	8	-3376.5	NA	-3378.3	-2565.9	NA	NA	-3364.1	-2590.3	NA	NA	-3382.1	-2541.3	NA	NA
	9	-3353.5	NA	-3317.1	-2559.4	NA	NA	-3327.9	NA	NA	NA	-3538.3	-2671.9	NA	NA

Covariance structures as described in Mclust v 5.4.8
*4 profiles with a VEV covariance structure was found to best fit the data

Supplementary Table S7. Classification probabilities to the four latent profiles

	Classification probability[a]							
	Profile 1		Profile 2		Profile 3		Profile 4	
Assigned profile	Mean	SD	Mean	SD	Mean	SD	Mean	SD
1	1.00	0.00	0.00	0.00	0.00	0.00	0.00	0.00
2	0.00	0.00	0.96	0.10	0.03	0.09	0.01	0.04
3	0.00	0.00	0.01	0.03	0.95	0.10	0.04	0.09
4	0.00	0.00	0.00	0.01	0.04	0.10	0.96	0.10

[a]Obtained from the z matrix returned from the Mclust command (Mclust package v5.4.8 in R)

Supplementary Table S8. WHODAS Impairment regressed on past mental disorder and current symptom severity and latent profiles (n=248)

	Model 1			Model 2		
	**Univariate Standardised beta (SE) [p]**	**Multivariate Standardised beta (SE) [p]**	**VIF**	**Univariate Standardised beta (SE) [p]**	**Multivariate Standardised beta (SE) [p]**	**VIF**
**Demographics**						
Sex (Male)	-0.16 (0.12) [0.19]	-0.01 (0.11) [0.948]	1.06	-0.16 (0.12) [0.19]	-0.06 (0.12) [0.597]	1.02
Age	0.01 (0.06) [0.82]	0.05 (0.06) [0.389]	1.06	0.01 (0.06) [0.82]	0.04 (0.06) [0.557]	1.03
**Past mental disorder**	0.48 (0.13) [<0.0001***]	0.11 (0.14) [0.407]	1.26	0.48 (0.13) [<0.0001***]	0.3 (0.14) [0.035*]	1.18
**Current symptom severity**						
Depression (PHQ9)	0.38 (0.06) [<0.0001***]	0.14 (0.11) [0.205]	3.77	-	-	-
Anxiety (GAD7)	0.36 (0.06) [<0.0001***]	0.01 (0.11) [0.9]	3.39	-	-	-
PTSD (PCL5)	0.32 (0.06) [<0.0001***]	0.05 (0.09) [0.567]	2.63	-	-	-
Somatization (PHQ15)	0.32 (0.06) [<0.0001***]	0.12 (0) [0.125]	2.01	-	-	-
SCI (AMIC)	0.22 (0.06) [<0.0001***]	-0.11 (0.08) [0.16]	1.97	-	-	-
Fatigue (CFQ)	0.38 (0.06) [<0.0001***]	0.23 (0.09) [0.017]*	2.92	-	-	-
**Latent profiles**						1.18
P1	-	-	-		ref	-
P2	-	-	-	0.05 (0.21) [0.80]	0.06 (0.21) [0.776]	-
P3	-	-	-	0.46 (0.19) [0.017*]	0.38 (0.19) [0.051]	-
P4	-	-	-	0.74 (0.22) [<0.0001***]	0.61 (0.22) [0.007**]	-
**Model building**						
Demographics	-	ref	-	-	ref	-
+ past mental disorders	-	F=13.89(1, 244) p<0.0001***	-	-	F=12.36(1, 244) p<0.0001***	-
+ current symptom severity	-	F=7.6(6, 238) p<0.0001***	-	-	-	-
+ latent profiles	-	-	-	-	F=3.78(3, 241) p=0.011*	-
**Model evaluation**						
R-squared	-	0.21	-	-	0.1	-
Adjusted r-squared	-	0.18	-	-	0.07	-
F-test	-	F=6.85(9, 238) p<0.0001***	-	-	F=4.26(6, 241) p<.0001***	-

PHQ9, Patient Health Questionnaire 9; GAD7, Generalized Anxiety Disorder 7; PCL5, Posttraumatic Stress Disorder Checklist for DSM-5; PHQ15, Patient Health Questionnaire 15; CFQ, Chalder Fatigue Scale; AMIC, Abbreviated Memory Inventory for Chinese; SCI, subjective cognitive impairment
*, **, *** = p<0.05, 0.01, 0.001

Figure legends

Supplementary Figure S1. Standardized mean scores from six self-rated scales across latent profiles. Error bars represent 95% confidence interval

PHQ9, Patient Health Questionnaire 9; GAD7, Generalized Anxiety Disorder 7; PCL5, Posttraumatic Stress Disorder Checklist for DSM-5; PHQ15, Patient Health Questionnaire 15; AMIC, Abbreviated Memory Inventory for Chinese; CFQ, Chalder Fatigue Scale

Supplementary Figure S2. Recruitment flowchart
