# Supplementary figures and images for: Dimensional structure of one-year post-COVID-19 neuropsychiatric and somatic sequelae and association with role impairment
Source: Sci Rep. 2023 Jul 27;13:12205. doi: 10.1038/s41598-023-39209-z (PMC10374659; doi:10.1038/s41598-023-39209-z)

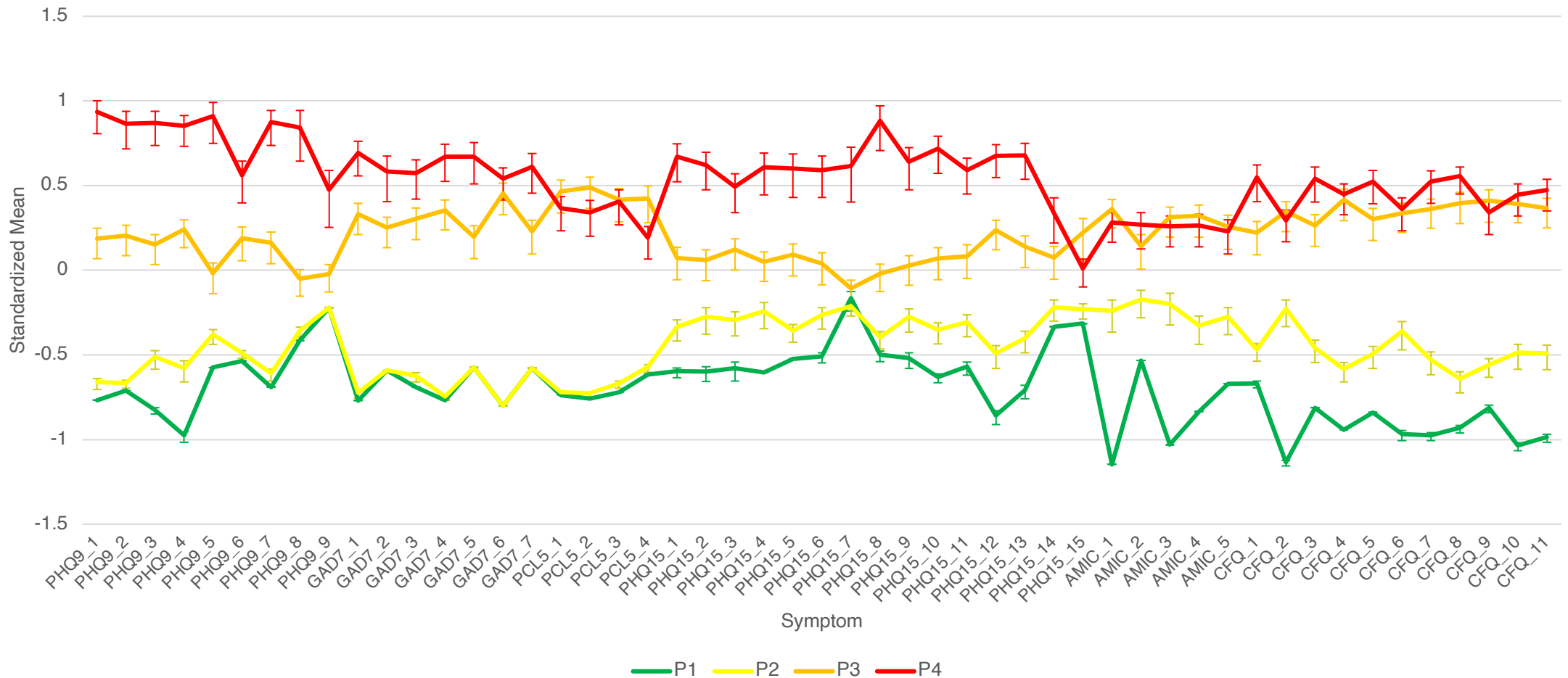

Supplement: Supplementary file 2 — Supplementary Figure S1. [file 41598_2023_39209_MOESM2_ESM.pdf]

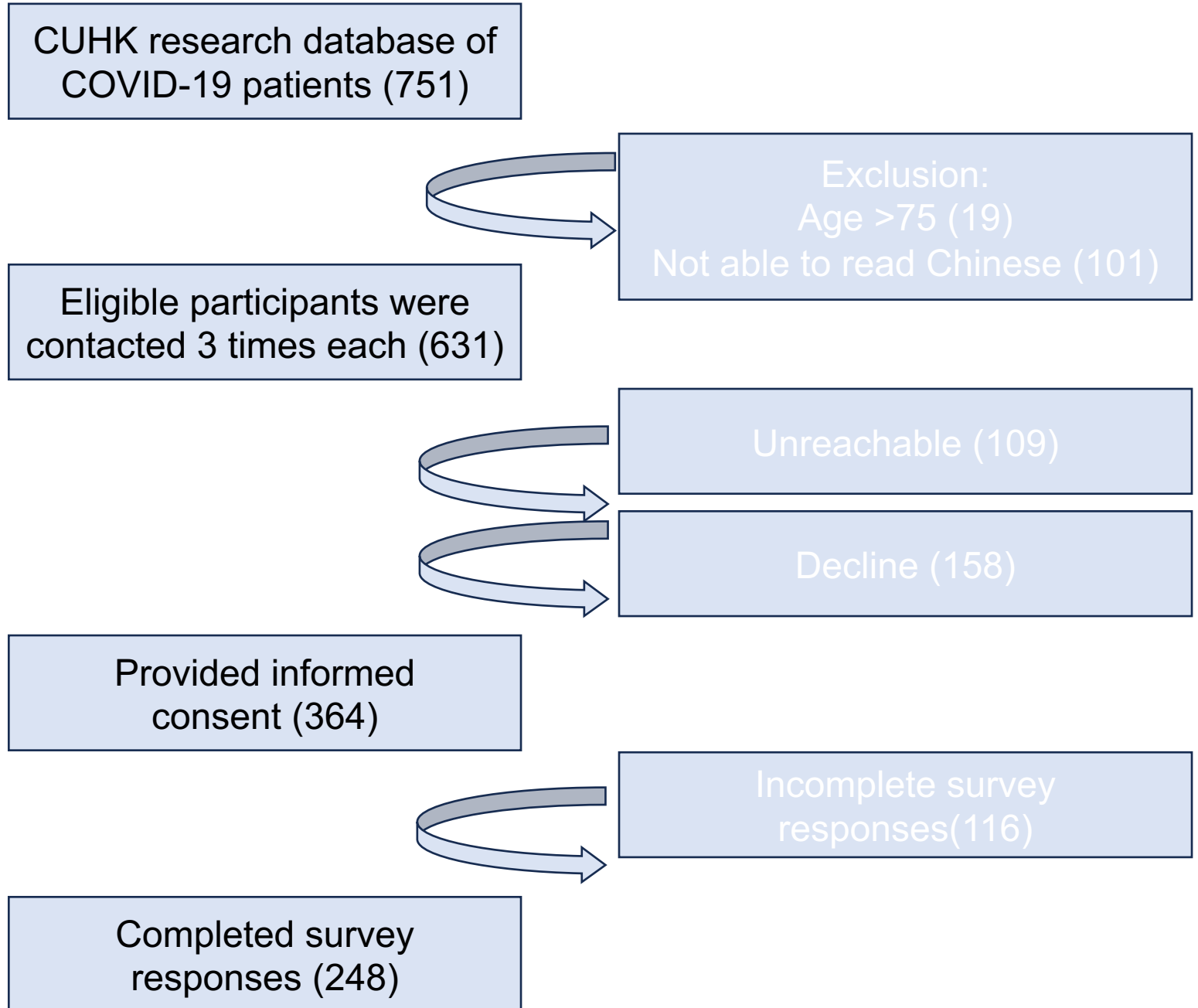

Supplement: Supplementary file 3 — Supplementary Figure S2. [file 41598_2023_39209_MOESM3_ESM.pdf]
